# Performance analysis and structural characterization of flaxseed gum/chitosan/cinnamaldehyde composite films

**DOI:** 10.1186/s13065-023-01054-6

**Published:** 2023-11-27

**Authors:** Xuejiao Ren, Na Wang, Xin Meng, Zhen Zhang

**Affiliations:** 1 College of Food and Health, Jinzhou Medical University, Jinzhou, China; 2 Innovation Center of Meat Processing and Quality Control Technology of Liaoning Province, Jinzhou Medical University, Jinzhou, China; 3 College of Food, Shenyang Agricultural University, Shenyang, China

**Keywords:** Flaxseed gum, Cinnamaldehyde, Composite film

## Abstract

The low mechanical strength, water deficiency, and oxidative protection of organic membranes impede their use as food-grade packaging materials. Cinnamaldehyde (CIN) tends to lose its activity owing to its instability. In this study, CIN was added to flaxseed gum (FG)/chitosan (CS) films prepared in a “sandwich” structure. The influence of CIN dosage on the properties of the composite films was studied, and the film formation mechanism of the membrane was explored. The elongation at break, water vapor permeability, oxygen permeability, and light transmittance of the composite film with 1.5% CIN were lower than those of the FG/CS/FG film. Supplementation of the composite membrane with CIN generated new hydrogen bonds, electrostatic interactions, and C-O-C bonds, which converted the structure of the composite film into a sheet and increased its crystallinity without markedly affecting its thermal stability. Therefore, CIN is an extremely useful additive for improving the applicability of flaxseed gum/CS membranes as food-grade packaging films.

## Introduction

Packaging plays an important intermediate role in extending the shelf-life of food and ensuring that food quality is not affected by the external environment [1]. Plastic is the most commonly used packaging material because of its light weight, low cost, excellent mechanical properties, and adequate stability [2]. However, chemically synthesized plastic packaging materials are non-biodegradable, which poses an immense risk to the environment. For example, discarded plastics in the ocean kill millions of marine lifeforms annually [3]. Research on food packaging materials has gradually shifted toward edible and biodegradable “green packaging” to prevent plastic pollution. The development of environment-friendly edible thin films will help reduce not only the use of plastic products but also carbon emissions [4].

Biodegradable packaging materials are edible materials that can be directly coated on food or cast into thin films. They are used as packaging materials to block contact between food and the external environment and inhibit microbial reproduction. In addition, the film matrix can be incorporated with nutrients to improve the nutritional content of food [5].

Flaxseed gum (FG) has high thermal stability, good oxidation resistance, and weak gel characteristics; thus, it is a suitable film-forming material. The application of FG to the preparation of edible films presents many advantages. FG is simple to extract [6], has a low generation cost compared with most biopolymers, is a natural renewable resource, and has good gas-barrier properties at low temperatures, which can extend the shelf life of food [7]. However, films made of FG have poor mechanical properties; moreover, the high viscosity, water solubility, and swelling degree of FG limit its applications in packaging materials. To compensate for the shortcomings of FG film formation, Hosseini et al. [8] developed an innovative method that uses biopolymers with complementary properties to prepare multilayer films and improve their material properties.

Chitosan (CS) is one of the most favored biopolymers today. It is biocompatible, biodegradable, and non-toxic. In addition, its transparency, UV resistance, and excellent oxygen-barrier properties render it a good film-forming material [9]. CS membranes have good barrier and mechanical properties and can reduce the spoilage caused by microorganisms in food.

Pure edible packaging membranes have virtually no biological activity, and their ability to extend the shelf life of food is limited. Therefore, active packaging composite membranes with high biological activity are urgently required. In recent years, many studies have focused on improving the bioactivity of edible packaging membranes by adding bioactive agents to improve the quality and extend the shelf life of food products [10].

Cinnamaldehyde (cassia bark aldehyde, CIN) has been widely used in many fields [11–13]. In the food industry, CIN is used in cakes, drinks, confectionery, and meat products, and serves as a preservative to inhibit the growth and reproduction of foodborne microorganisms such as *Staphylococcus aureus*. However, CIN has a strong smell, and its direct addition to food can have adverse sensory effects. Moreover, CIN is easily volatilized and oxidized during storage, leading to a significant decrease in its antibacterial activity [14].

Active packaging designs with a sandwich structure can be used to control the release quantity of CIN and increase its duration of action. This type of packaging material is typically composed of three different layers: the barrier, active, and control layers [15]. The barrier layer, as the outermost layer of the laminated structure, is in direct contact with the external environment. It is often designed to block substances that degrade packaged foods, such as water, oxygen, and microorganisms. The barrier layer can also be designed to ensure the good retention and protection of the active ingredients within the packaging material. The active layer is located in the middle of the sandwich-like structure and usually contains active components such as antioxidants and antimicrobials [16]. Ensuring that the active ingredients remain in the active layer and do not diffuse through the control layer to the food until required is important. The control layer, as the innermost layer of the sandwich-like structure, is in direct contact with food. It is designed to control the rate at which active ingredients enter food to protect both the food and active ingredients. Several studies have shown that the release of active components can be controlled by tuning the structure of the multilayer packaging, thereby extending the duration of action. For example, a multibiodegradable film could be prepared by the layered solvent casting of zeol as the barrier layer, tea polyphenols as the active layer, and gelatin as the control layer [17]. The water separation properties and release time of tea polyphenols can be controlled by changing the ratio of zeolmin/gelatin in the active layer. The release time of tea polyphenols in the multilayer system was longer than that in the control group.

In this study, CS and CIN were added to FG films. The film was structurally characterized and its formulation was optimized to improve its performance. The mechanisms responsible for film formation and antibacterial activity were studied in detail to determine a formulation for a packaging material with excellent preservation capabilities.

## Materials and methods

### Materials

Flaxseed was procured from Xinghe County, Wulanchabu, Inner Mongolia. CS (deacetylation > 95%) and CIN were purchased from Shanghai Aladdin Biochemical Technology Co., Ltd. The glycerol and acetic acid used were of analytical reagent grade.

### Preparation of FG

The FG used in this work was extracted using the method developed by Ren et al. [18]. Briefly, the flaxseed was soaked in distilled water at a flaxseed-to-water ratio of 1:15 (w/w) at 60 °C and 240 W under continuous and gentle stirring for 30 min in an ultrasonic-assisted extraction water bath. The resulting solution was centrifuged at 4000 rpm for 10 min and treated with three times its volume of 95% ethanol to precipitate the gum. The precipitated gum was centrifuged at 4000 rpm for 10 min, dried in a hot air oven at 50 °C, and stored at 4 °C.

### Preparation of FG/CS/CIN films

The films were prepared using a method developed by Yang et al. [19] with slight modifications. FG (0.50 g) was completely dissolved in 100 mL of deionized water and stirred at 60 °C. Glycerol (20% v/w, FG) was added, and the mixture was stirred at 60 °C in a water bath for 30 min and stored overnight at room temperature. The resulting solution was labeled “FG film solution.” CS (1.5 g, 95% deacetylation) was added to 100 mL of 1% (v/v) glacial acetic acid solution and stirred in a water bath maintained at 60 °C. Glycerol (20% v/w, CS) was added, and the mixture was stirred at 60 °C in a water bath for 30 min. The resulting solution was labeled “CS film solution.” Tween-80 (0.5% v/v) was added to the CS film solution, and the mixture was magnetically stirred for 30 min at 20 °C. Finally, CIN was added at ratios of 0.5%, 1.0%, 1.5%, and 2.0% (v/v), and the mixture was stirred for 30 min to form a CS-CIN film solution.

The FG film solution (10 mL) was poured into a disposable sterile plastic flat plate (diameter, 90 mm), cast evenly, and dried at 40 °C until slightly dry and viscous to obtain the first layer. The CS film solution (10 mL) was poured over the first layer and dried at 40 °C until slightly dry and viscous to obtain the second layer. Then, 10 mL of the FG film solution was poured over the second layer under the same conditions used for the first layer. Finally, the FG/CS composite film was obtained. The “sandwich” FG/CIN/CS composite film was prepared with the CS-CIN film solution instead of the CS film solution.

### Film thickness

The thickness of the films was determined using an electron number thickness gauge (SYNTEK-59, Deqing Shengtai Core Electronic Technology Co., Ltd., China). Nine points were randomly selected at the four corners and center of the membranes, the thickness of the films at these nine points was measured (unit: mm) using a handheld helical microscope, and the results were averaged [20].

### Mechanical properties

The tensile strength (TS) and elongation at break (EB) of the film were measured by using a physical test instrument (TA.XT.Plus, SMS Co., UK) in accordance with the procedures specified by Liu et al. [21]. After softening in a low-temperature climate box, the film was cut into a 60 mm × 15 mm rectangle and fixed on the probe. The rate of elongation and total elongation were set at 5 mm/s and 50 mm, respectively. The probe gradually stretched the membrane to failure and recorded the stress–strain data. Each sample was tested three times, and the results were averaged. TS and EB were calculated as follows:

$${\rm{TS(MPa}}) = \frac{{{\rm{F}} \times {{10}^{ - 6}}}}{{\rm{S}}}$$

where *F* is the membrane fracture load at the maximum tension (N) and *S* is the cross-sectional area (m^2^) of the sample, and

$${\rm{EB(\% )}} = \frac{{{{\rm{L}}_1}{\rm{ - }}{{\rm{L}}_0}}}{{{{\rm{L}}_0}}} \times 100{\rm{\% }}$$

where *L*_*1*_ is the fracture length of the membrane (mm) and *L*_*0*_ is the initial length of the membrane (mm).

### Water vapor permeability (WVP)

The WVP of the film was measured by the cup-fitting method [22]. The silica particles and conical flasks were dried at 105 °C in an oven and then cooled to 25 °C. The mouths of the flasks were completely sealed using sealing film. Next, the conical flasks were placed inside a dryer containing distilled water to maintain 100% relative humidity. The flasks were removed from the dryer after 12 h and weighed, and the time of weighing was accurately recorded for 7 d. The WVP of the film was calculated by determining the increase in the mass of the conical flask. Three samples were collected from each group, and the WVP (g·mm/h·m^2^·kPa) was calculated according to the following equation:

$${\rm{WVP}} = \frac{{\Delta {\rm{m}} \times {\rm{d}}}}{{\Delta {\rm{t}} \times {\rm{A}} \times \Delta {\rm{p}}}}$$

where Δ*m* is the mass increment (g), *d* is the film thickness (mm), Δ*t* is the interval between two weighing instances (h), *A* is the area of the sample covering the mouth of the flask (m^2^), and Δ*p* is the saturated vapor pressure of pure water at the testing temperature (2.861 kPa).

### Oxygen permeability (OP)

The OP of the film was determined by the differential pressure method according to ASTM D1434-82 (2015). The testing temperature was 23 °C [23].

### Light transmittance (LT)

The LT of the films was determined using a UV-Vis spectrophotometer (UV2500, Shimadzu Co., Japan) at 600 nm. The film samples were trimmed to a 50 mm × 10 mm rectangle close to the inner surface on the side of the quartz cuvette, and a blank cuvette was used as the control.

### Attenuated total reflectance Fourier-transform infrared (ATR-FTIR) spectroscopy

The composite membrane was subjected to ATR-FTIR spectroscopy using an FTIR spectrometer (Nicolet is5, Thermo Fisher Scientific, Waltham, MA, USA) at a spectral resolution of 4 cm^− 1^ using 32 co-added scans in the wavenumber range of 4000–400 cm^− 1^.

### Scanning electron microscopy (SEM)

The membrane was cut into a circle with a diameter of 55 mm and affixed to the copper platform of the SEM instrument (GeminiSEM 500, ZEISS, Jena, Germany) using conductive glue. Gold spray-coating was performed prior to observing the microstructure of the film at an accelerating voltage of 5 kV.

### X-ray diffraction analysis

The crystal structure of the film was determined using a diffractometer (D8 Advance, Bruker, Billerica, MA, USA). Cu Kα radiation (1.542 Å) was generated at an accelerating voltage of 40 kV and a current of 40 mA. The diffraction angle (2*θ*) range was 3°–60°, the scan step size was 0.02°, and the scan speed was 4 min^− 1^.

### Thermodynamic property analysis

The method developed by Kumar et al. [24] was used with minor modifications. A 5.0 mg composite film sample was subjected to thermogravimetric (TG) analysis under a nitrogen environment at a heating rate of 10 °C/min in the temperature range of 30–500 °C. Additionally, 5.0 mg of the composite film was weighed in aluminum sample discs for differential scanning calorimetry (DSC), with empty aluminum discs used for reference. All DSC measurements were performed at a heating rate of 10 °C/min using nitrogen supplied at a flow rate of 50 mL/min as the carrier gas to analyze the heat flow changes of different samples in the temperature range of 30–500 °C.

### Statistical analysis

The variance and its significance were analyzed using SPSS Statistics 22.0 (IBM, USA) and Origin 2016 (OriginLab, USA), and the data were analyzed for differential significance using analysis of variance. All data are recorded as the average of parallel experiments, and the results are expressed as mean ± standard deviation.

## Results and discussion

### Effect of CIN dosage on the mechanical properties of the composite film

**Fig. 1:**
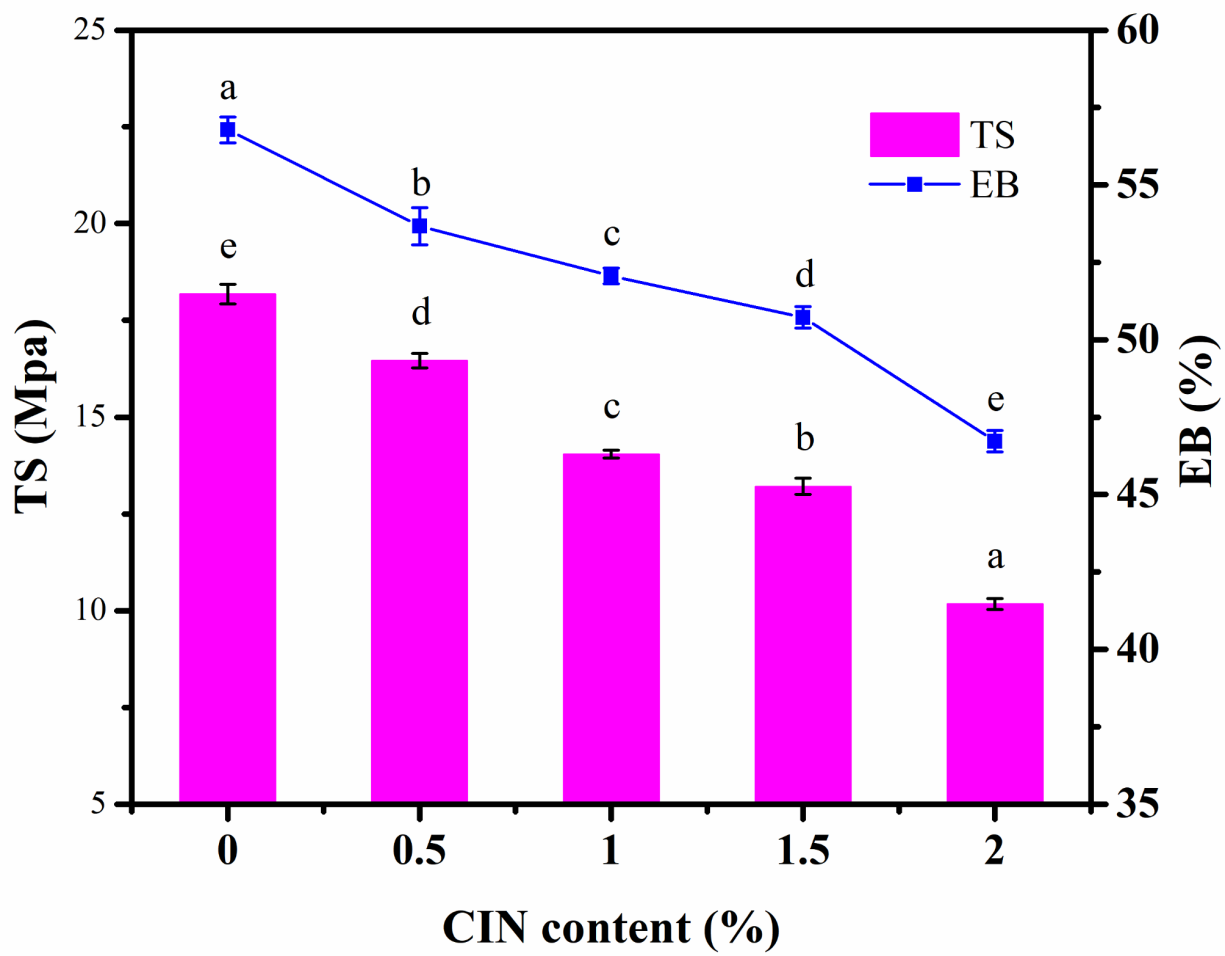
Mechanical properties of flaxseed gum (FG)/cinnamaldehyde (CIN)/chitosan (CS) composite films. Different letters represent significant differences (*p* < 0.05)

The effect of CIN addition on the mechanical properties of the composite film is shown in Fig. 1. Unlike that of the FG/CS film, the TS of the FG/CIN/CS composite film decreased significantly following the addition of CIN. The dosage of CIN influenced the mechanical properties of the composite membrane significantly (*p* < 0.05). The presence of CIN destroyed the tight structure of the CS and FG membranes, increased the spacing and reduced the entanglement between FG and CS molecular chains, and weakened the tensile strength of the composite films [25]. These changes led to the arrangement of the CIN/CS film layers into a lamellar stacking pattern, and the structural discontinuity within the polysaccharide network led to reductions in TS and EB. Moreover, the water content in the film decreased with increasing hydrophobic CIN content. The interaction of water with the majority of the hydrophilic colloidal substances changed the structure of the natural polymer and, in turn, the mechanical properties of the film. Jahed et al. [26] found that the addition of fennel essential oil to CS could significantly improve the TS and reduce the EB of the membrane. By contrast, Hosseini et al. [27] found that the addition of clove and thyme essential oils (TEOs) to CS significantly reduced the TS of the composite membrane and increased its EB.

### Effect of CIN dosage on the WVP of the composite film

**Fig. 2:**
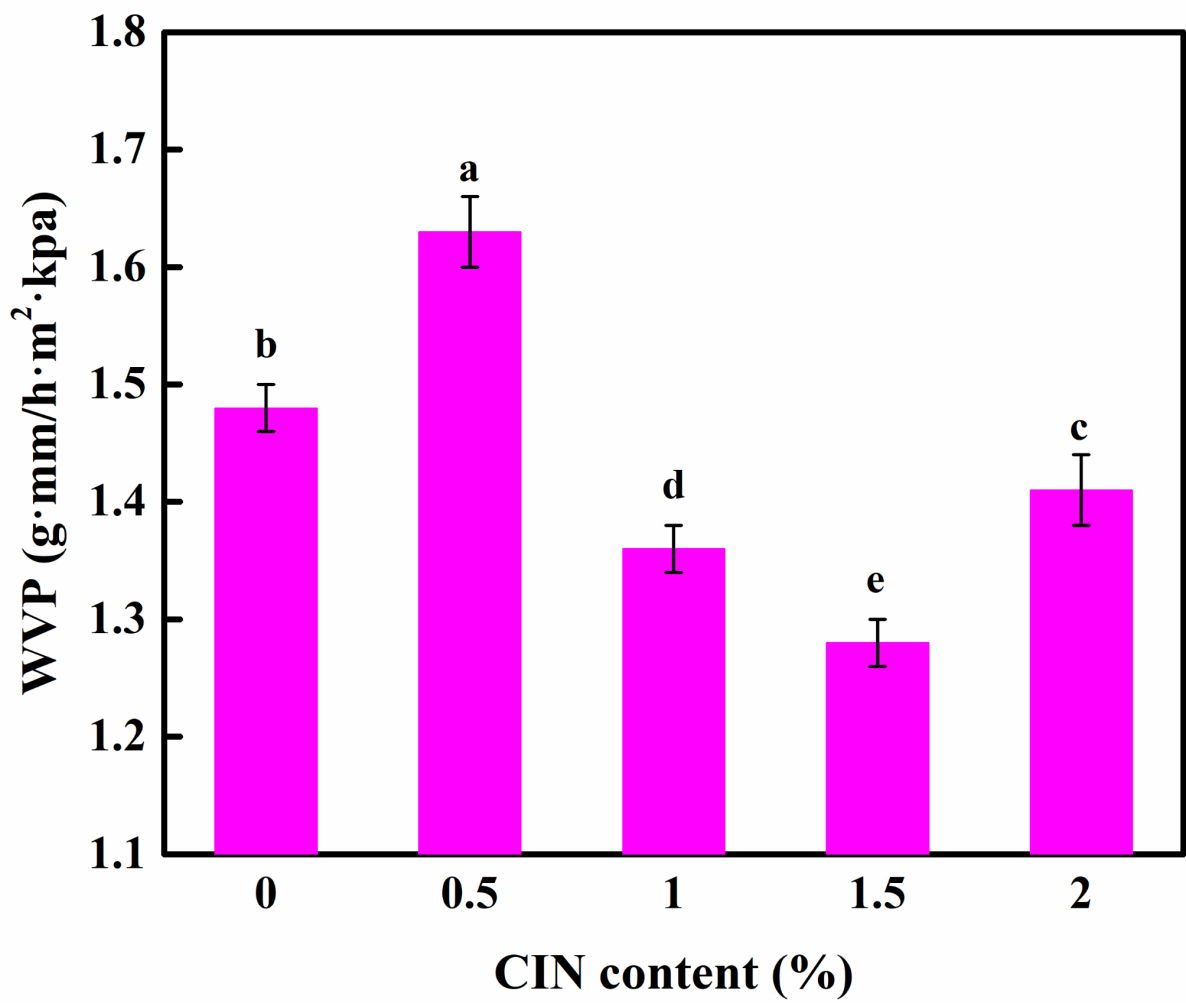
Water vapor permeability of the FG/CIN/CS composite films. Different letters represent significant differences (*p* < 0.05)

The effect of CIN addition on the WVP is shown in Fig. 2. With increasing CIN content, the WVP initially increased, subsequently decreased, and then increased once more. When the quantity of CIN added was less than 0.5%, the CIN molecules diffused into the composite membrane and dispersed uniformly in the polymer chains, thereby breaking the dense structure of the composite membrane, creating holes and cavities, facilitating the passage of water vapor, and increasing the membrane permeability. It hints that CIN more of a physical absorption on the film than being a scavenger. When the quantity of added CIN was increased to 1.5%, CIN was uniformly dispersed in the macromolecular network structure owing to its hydrophobic properties, thereby increasing the water transfer bending factor of the membrane matrix and the distance through which water molecules diffuse in the film. These phenomena could effectively prevent the passage of water molecules and reduce the WVP. As the quantity of CIN added was increased to 2.0%, a surplus of CIN destabilized the composite membrane structure, leading to the agglomeration of CIN, void creation inside the membrane, and water passage through the membrane. Zhang et al. [28] prepared a TEO nanoemulsion gel CS multilayer film and showed that the WVP increased with the addition of TEO.

### Effect of CIN dosage on the OP of the composite film

**Fig. 3:**
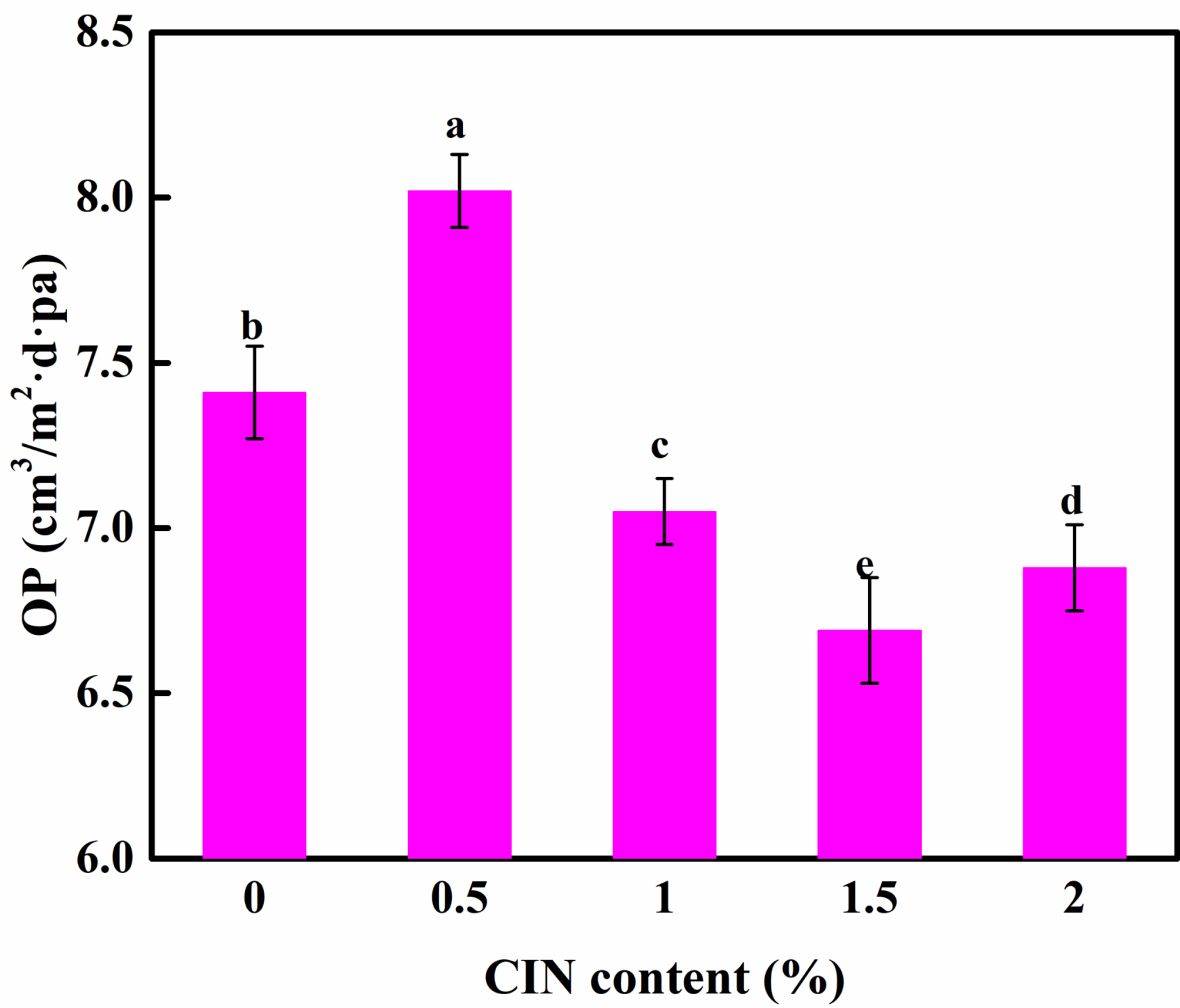
Oxygen permeability of FG/CIN/CS composite film. Different letters represent significant differences (*p* < 0.05)

Oxygen is the main environmental factor responsible for the deterioration of food during storage. As shown in Fig. 3, as the concentration of CIN increased from 0 to 0.5%, the OP increased, indicating that CIN molecules are buried in the matrix and do not work as O_2_ scavenger at low concentration. According to the SEM images, the oxygen-isolating capacity of the film decreased owing to the volatility of CIN, which generated more pores inside the film. When the dosage of CIN was increased from 0.5 to 1.5%, the OP gradually decreased to less than that of the control group, indicating that the oxygen-isolating ability of the film gradually increased. This finding may be related to the emulsification of Tween-80, which enhances the preservation of CIN in the film and prevents phase separation in film systems. The compatibility between FG, CS, and the essential oil rendered the film structure more compact, thereby preventing the entry of nonpolar O_2_ molecules into the film. In addition, CIN has high antioxidative activity, scavenges free radicals, and reduces the oxygen transmittance of the film. When the CIN content reached 2.0%, CIN agglomeration occurred, thereby disrupting the balance of the structure, creating voids inside the membrane, and increasing the OP.

### Effect of CIN dosage on the LT of the composite film

**Fig. 4:**
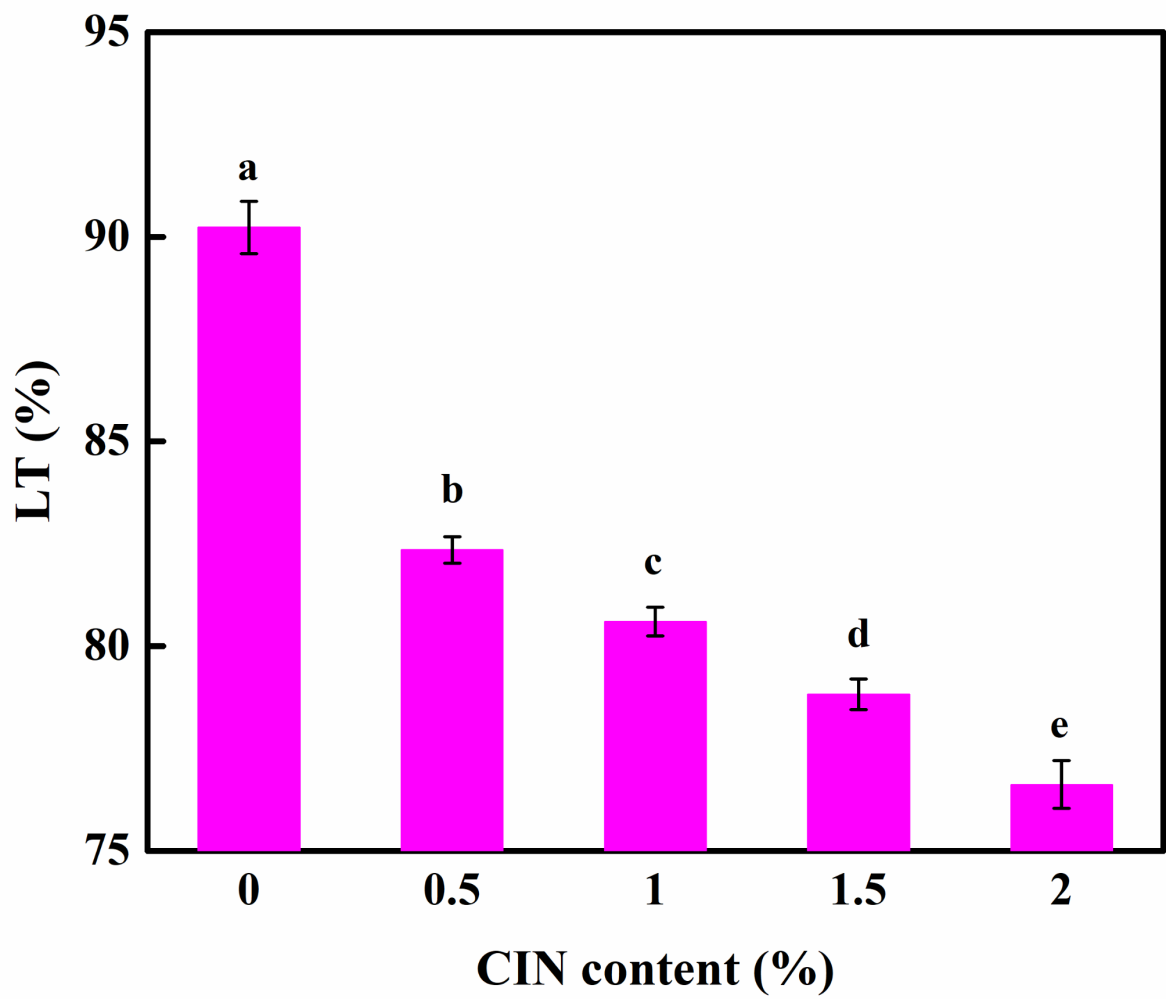
Light transmittance of FG/CIN/CS composite film. Different letters represent significant differences (*p* < 0.05)

The effect of different quantities of CIN on the LT of the composite film is shown in Fig. 4. The highest LT was recorded in the absence of CIN, indicating that, among the films, the transparency of the FG/CS film was the highest. When the CIN content was increased from 0.5 to 2.0%, the LT of the film decreased significantly (*p* < 0.05). The number of cavities within the film structure may have increased owing to the destructive effect of CIN on the CS crystal network, and the addition of hydrophobic CIN to the composite membrane can promote the scattering of light at the oil droplet interface. In addition, the color of CIN may reduce the LT of the film by reducing its transparency. Although in terms of appearance, the transparency of the film is an important factor affecting consumer psychology, the film dosed with CIN can effectively prevent the passage of ultraviolet light, and the packaging could facilitate the antioxidative protection of photosensitive food [29].

### Effect of CIN dosage on the intermolecular forces in the composite film

**Fig. 5:**
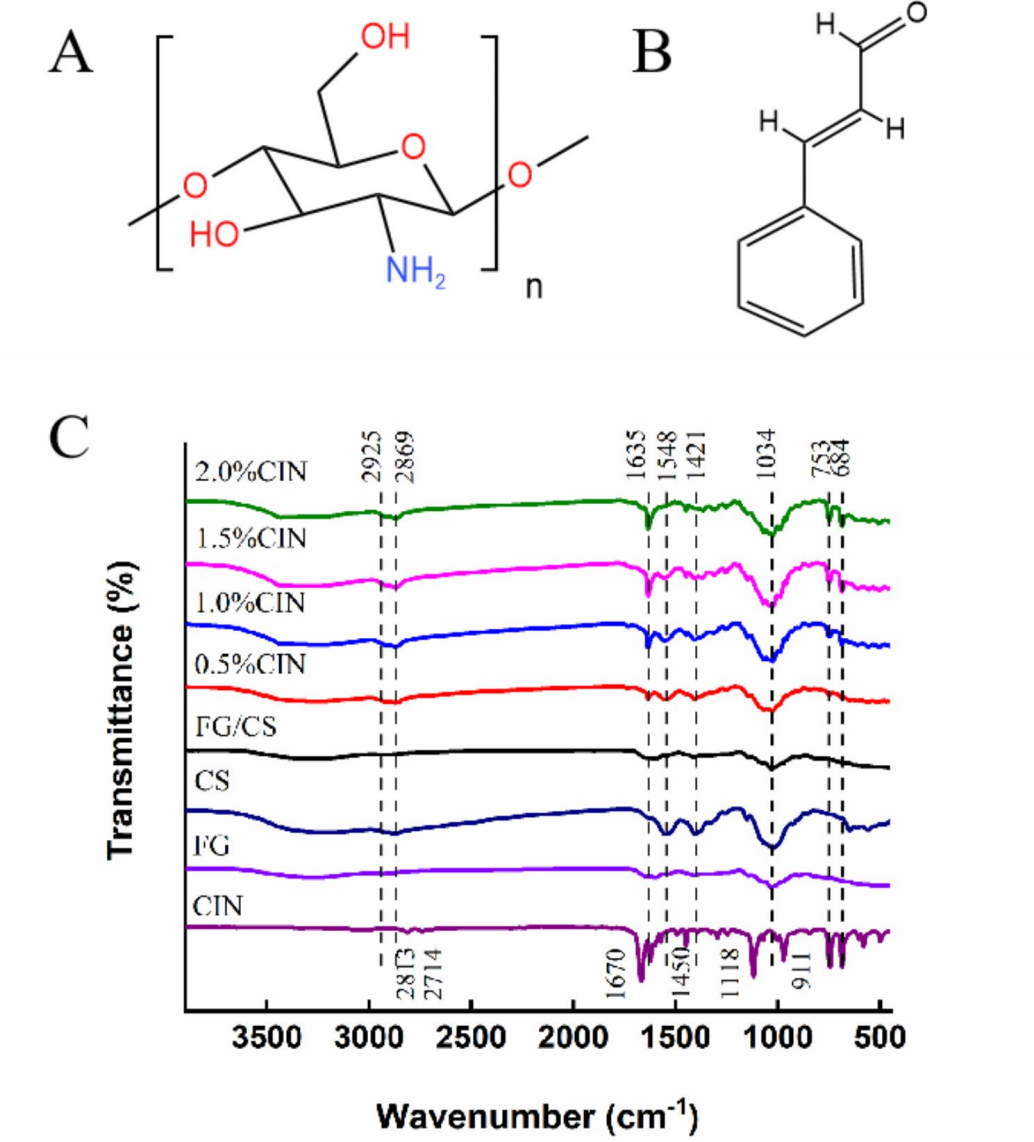
Fourier-transform infrared spectra of each component and composite film. (A) Chitosan. (B) CIN. (C) ATR-FTIR.

To study the interaction between membrane molecules, we performed ATR-FTIR characterization of the film-forming raw materials and membrane samples. As shown in Fig. 5, the broad absorption peaks of CS and FG at 3050–3606 cm^− 1^ can be attributed to the telescopic vibrations of intermolecular and intramolecular O-H, the weak absorption peaks at 2925 and 2869 cm^− 1^ can be attributed to the symmetric and asymmetric telescopic vibrations of C-H, and the absorption peak of CS at 1548 cm^− 1^ can be attributed to the telescopic bending vibrations of N-H. The absorption peak near 1412 cm^− 1^ reflects the telescopic vibrations of C-N, and the absorption peak at 1034 cm^− 1^ reflects the symmetrical expansion and contraction of C-O-C. The CIN peaks at 2813 and 2714 cm^− 1^ are the C-H telescopic vibrations of the aldehyde group [30], and the characteristic absorption peak at 1670 cm^− 1^ represents C = O.

During the formation of antibacterial films, the displacement of the broad absorption peak at 3050–3606 cm^− 1^ in the long-wave direction indicates that a new H bond is formed in the reaction, and the H bond strength increases with increasing quantity of CIN. The absorption peaks at 2925 and 2869 cm^− 1^ significantly increased in size with the gradual increase in CIN content, indicating that the R-CHO in CIN combined with conjugated double bonds in CS and FG during the film-forming process, producing electrostatic interactions. The peak at 1635 cm^− 1^ could be attributed to C = N bonds, thus proving that a Schiff base was formed during the reaction [31]. The absorption peak of CS at 1548 cm^− 1^ decreased in size, and CIN film formation gradually decreased, indicating that the Maillard reaction and formation of a Schiff base during film formation resulted in the consumption of N-H. The increase in absorption peak intensity at 1034 cm^− 1^ was due to the formation of new C-O-C cyclic ether bonds. This finding indicates that new chemical bonds are generated after the addition of CIN and that these bonds affect the performance of the prepared antimicrobial film. The results suggest that these new forces limit motion between the molecules, thereby altering the physical properties and gas-barrier performance of the composite membrane.

### Effect of CIN dosage on the microstructure of the composite film

**Fig. 6:**
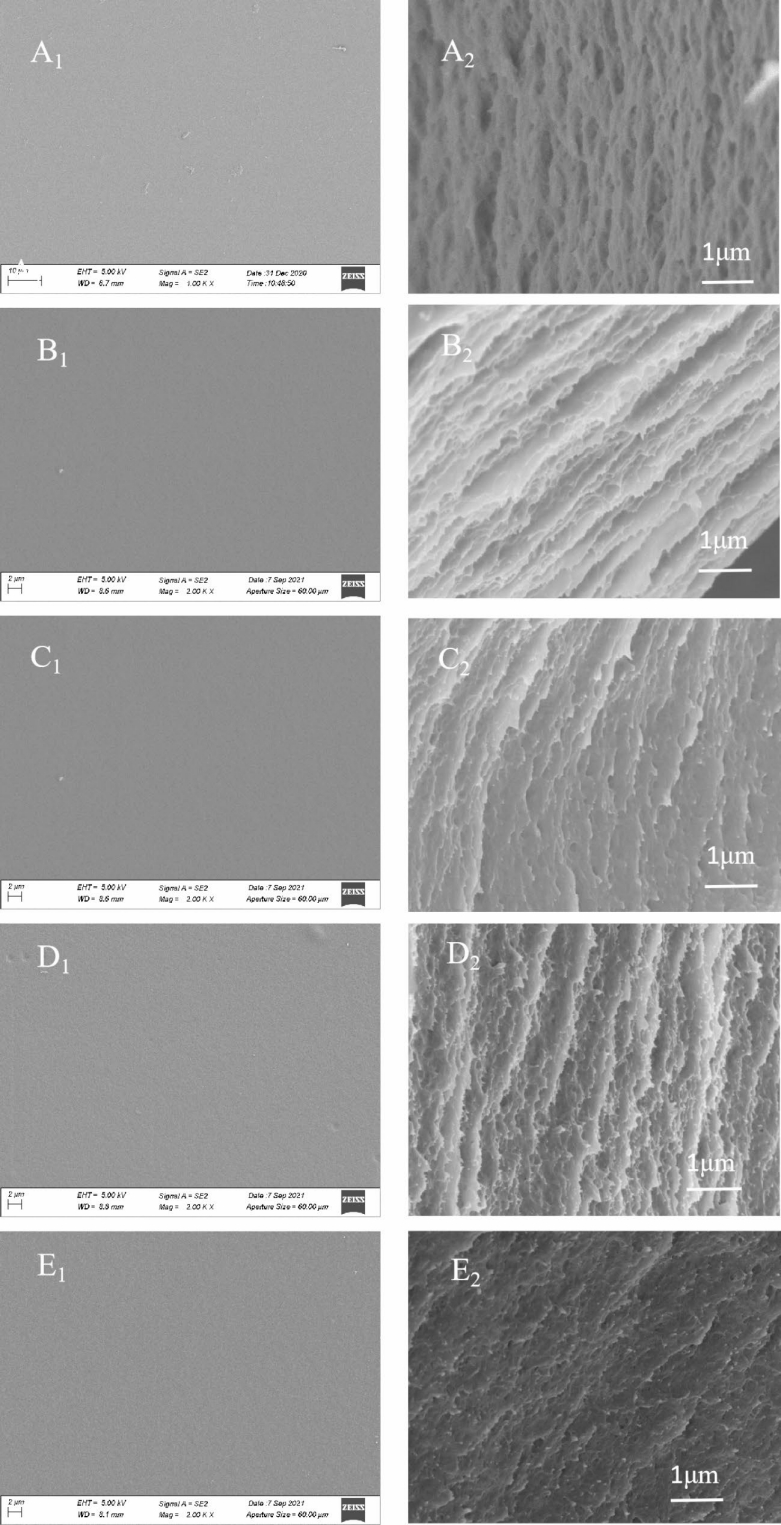
Scanning electron microscopy images of the FG/CIN/CS composite film. (**A**) FG/CS, (**B**) 0.5% CIN, (**C**) 1.0% CIN, (**D**) 1.5% CIN, and (E) 2.0% CIN. A_1_–E_1_ depict the surface structures, and A_2_–E_2_ depict the cross-sectional structures of the membranes

The effect of CIN concentration on the microstructure of the composite film was observed using SEM (Fig. 6). By comparing the superficial and sectional structures of the membrane, we found that the surfaces of all films were uniform, smooth, and uncracked, which indicates that the components fused together well. The cross-section of the FG/CS film exhibits a dense network structure. When over 0.5% (v/v) CIN is added, the cross-section appears as a layered sheet structure that is full of holes, which may be caused by a change in the network structure of the film or changes in the network structure of the membrane owing to the volatilization of essential oils and hydrophobic interactions between essential oils and polysaccharide groups during film formation. When the quantity of CIN added was increased to 1.0–1.5%, the layered sheet structure gradually disappeared and the film became scaly, indicating that CIN promoted the tight combination of the surrounding CS and FG polysaccharides at these concentrations. As the CIN concentration continued to increase to 2.0%, white spots were produced owing to the agglomeration of essential oils, indicating that a high concentration of CIN destroys the network structure between CS and FG, resulting in a significant decrease in the EB of the composite membrane.

### Effect of CIN dosage on the crystallinity of the composite film

**Fig. 7:**
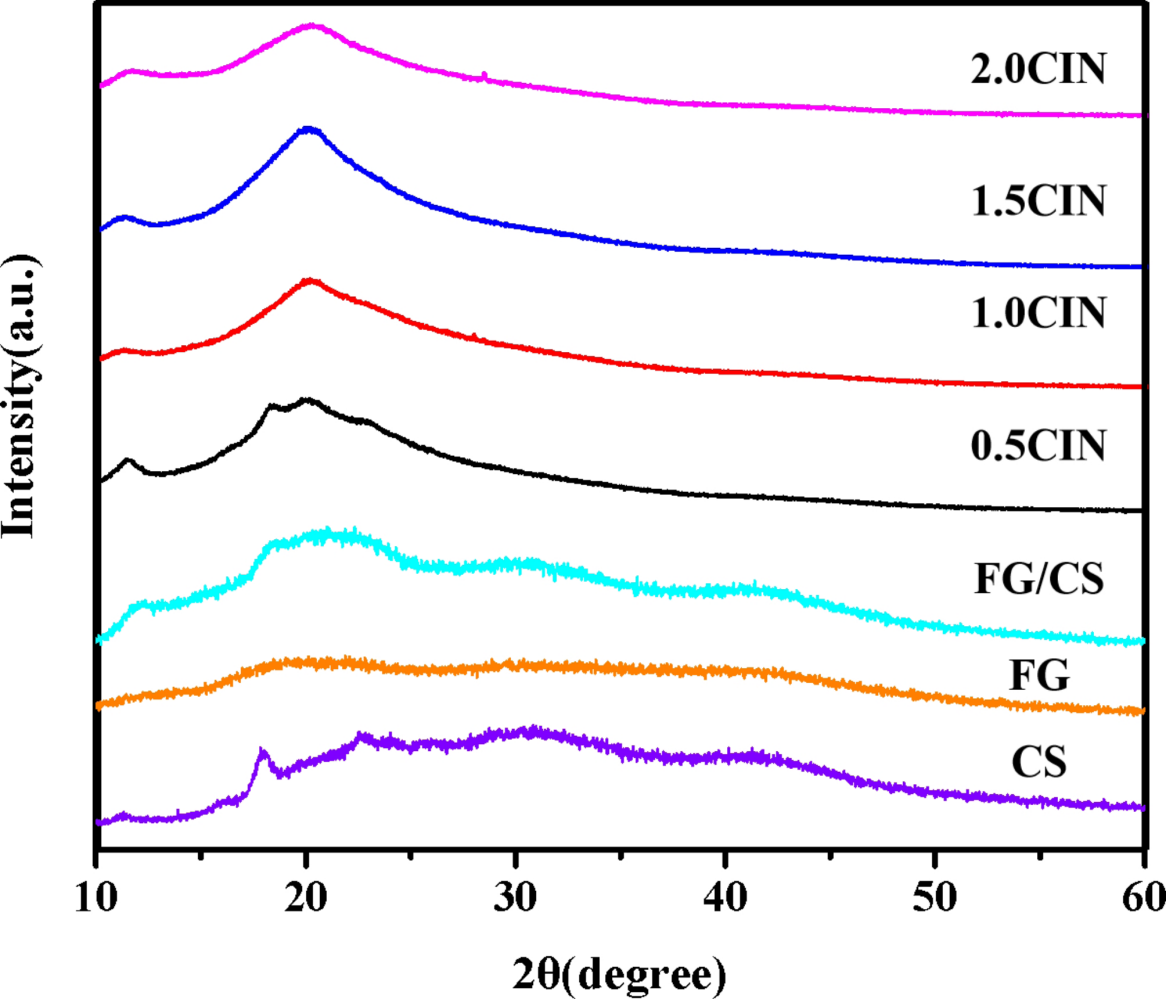
X-ray diffraction patterns of the FG/CIN/CS composite film

As shown in Fig. 7, three diffraction peaks were observed at approximately 2θ = 11.26°, 17.89°, and 22.56°, indicating that CS had obvious crystalline regions. FG did not produce any diffraction peaks, indicating that it exists in a non-crystalline form. After the formation of the FG/CS composite film, all three sharp diffraction peaks of CS disappeared, indicating that the interactions generated during the formation of the composite film destroyed the crystalline morphology formed by the regular arrangement of CS. Following the addition of CIN, significant diffraction peaks appeared in the vicinities of 2*θ* = 11.36° and 19°, indicating that the crystallinity of the composite membrane increased compared with that of the control group. This phenomenon may be attributed to the interactions of CIN with CS and FG to form new crystallization regions that increase the crystallinity of the film. However, when the CIN content was increased to 2.0%, the crystallization region began to shrink, probably because of the disruption of intermolecular interactions caused by hydrophobic CIN. Furthermore, the disappearance of the broad peaks in the FG/CS film at 30.45° and 41.38° indicated the transition of the FG/CS membrane from the amorphous state to the crystalline state following the addition of CIN.

### Effect of CIN dosage on the thermodynamic properties of the composite film

**Fig. 8:**
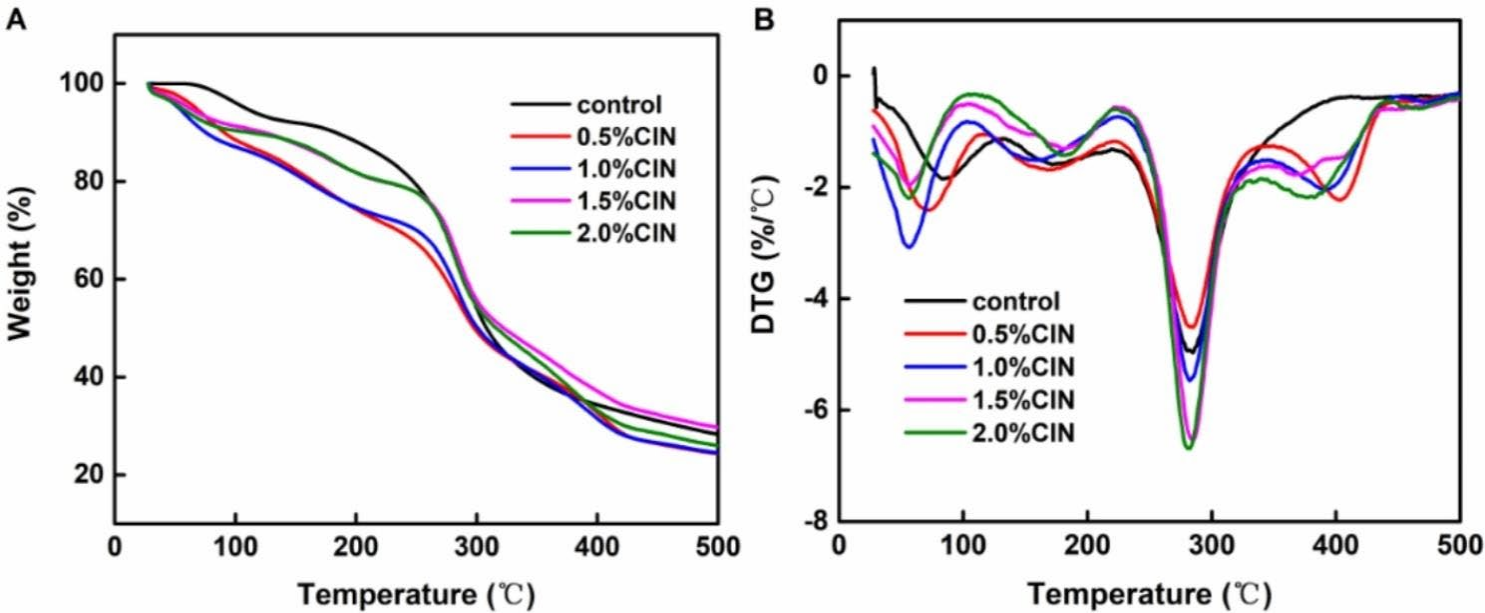
Thermogravimetric analysis/differential TG results for the FG/CIN/CS composite film

The thermal stability of the composite membrane was evaluated by TG analysis. The TG weight-retention profile and differential TG profile of the FG/CS (control) and CIN (0.5%, 1.0%, 1.5%, and 2.0%) films are shown in Fig. 8; Table 1. According to the TG profile (Fig. 8A), the weight-loss curve of the control group was relatively gentle, and the extent of mass loss was 71.75%. As the CIN content increased to 0.5%, 1.0%, 1.5%, and 2.0%, mass losses of 75.67%, 75.50%, 70.29%, and 74.04%, respectively, were recorded. In addition to the evaporation of water, this change is also related to the decomposition of FG and CS. The thermal decomposition of CIN may further affect the thermal stability of the composite membrane.

As shown in Fig. 8B, the differential TG profile of the composite membrane dosed with CIN differed from that of the control group. In the control group, the initial weight-loss stage ranged from 0 to 130 °C and was predominantly attributed to the evaporation of water and decomposition of small molecules such as acetic acid [32]. The second weight-loss stage ranged from 130 to 220 °C and was predominantly attributed to the degradation of FG and CS side-chain pyran rings [26]. The third weight-loss stage ranged from 220 to 400 °C and was attributed to the disintegration of FG/CS molecular chains [33]. Following the addition of CIN, the composite membrane exhibited four weight-loss peaks in the range of 0–110 °C, which is a slightly lower temperature range relative to that of the control group. These peaks could be attributed to the evaporation of water and decomposition of small molecules such as acetic acid, which may have occurred because of the disruption of the chemical bonds between the hydrophilic substances and water molecules following CIN addition, thereby facilitating the release of water molecules. The second weight-loss stage fell within the temperature range of 110–220 °C and was attributed to the decomposition of glycerol. Relative to that of the control, the decomposition temperature of the composite membrane increased slightly following the addition of CIN, possibly because of the generation of a Schiff base between CIN and CS during film formation and the addition of Tween-80, which had a good emulsification effect and increased the degradation temperature. The lower degradation temperature of films with CIN (0.5% and 1%) compared with that of the control (FG/CS) may be attributed to the presence of CIN, which destroys the balanced structure of CS, FG, and glycerol. The third weight-loss stage fell between 220 and 340 °C and was mainly attributed to the degradation of CIN molecular chains in FG/CS/CIN. The addition of CIN had an insignificant effect on the maximum degradation temperature of the composite membrane. Stage 4, ranging from 340 to 440 °C, was not observed for the control group and was related to the breakdown of CIN. In addition, a small weight loss peak was observed at approximately 465 °C, which may be attributed to the presence of residual Tween-80.

**Table 1:** Results of the thermogravimetric analysis of the FG/CS/CIN composite film

Sample	Step 1 (°C)	Step 2 (°C)	Step 3 (°C)	Step 4 (°C)	Residue at 500 °C (%)
FG/CS	80.9	174.8	283.2		28.25
0.5% CIN	67.6	168.6	286.6	399.5	24.33
1.0% CIN	53.6	156.1	284.1	392.5	24.50
1.5% CIN	58.8	185.1	284.6	406.0	29.71
2.0% CIN	54.1	184.6	283.1	378.5	25.96

The maximum degradation temperature of the CIN composite membrane at different concentrations was not significantly different from that of the control group. Thus, we can infer that CIN has no significant effect on the thermal stability of the composite membrane, which mainly depends on FG/CS composite formation.

### Proposed mechanism for membrane formation

**Fig. 9:**
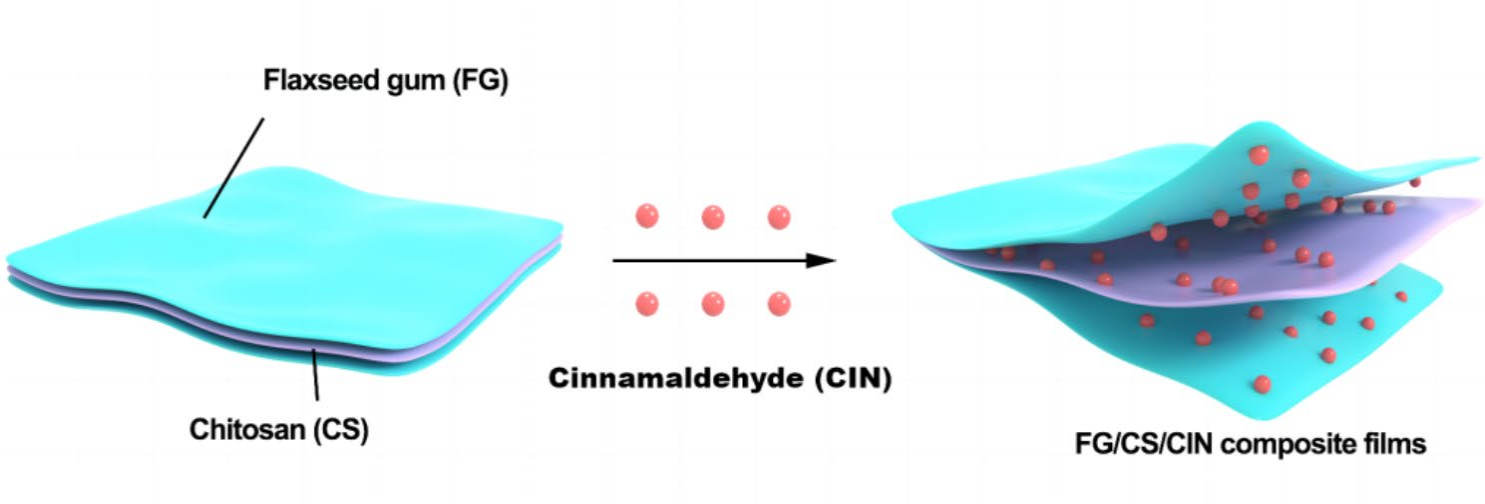
Schematic diagram showing the mechanism of FG/CIN/CS composite film formation

The membrane formation process was inferred from the performance index and structural characterization of the FG/CS/CIN composite membrane and is shown in Fig. 9. CS is a positively charged polysaccharide, and the zeta potential of FG is − 12.3 ± 1.22 mV [14]. Therefore, FG and CS molecules can interact electrostatically. FG contains glyonic acid. Therefore, the -COOH or -OH groups present in its side chain and the -NH_2_ groups present in the CS molecules can interact by H-bonding, thereby increasing the attraction between FG and CS molecules as well as the density of the film.

The ATR-FTIR spectra indicate that after the addition of 0.5% CIN to the composite membrane, new electrostatic interactions and -C-O-C- bonds were formed between the molecules, limiting intermolecular movement. As a small molecule, CIN diffuses into the FG/CS composite membrane and is uniformly dispersed in the polymer chain, thereby breaking the dense structure of the composite membrane and creating cavities that facilitate the passage of gases and increase the WVP and OP.

When the CIN content was increased to 1.5%, the optimum balance between various forces was attained. The ATR-FTIR spectrum indicates that the formation of a new H-bond between CS and CIN increases the attraction between the film molecules. Enhancements in electrostatic interactions and -C-O-C- bonds improved the surface structure, TS, and EB of the composite film. Owing to its hydrophobicity, CIN is uniformly dispersed in the macromolecular network, thereby increasing the bending factors for water transfer in the membrane matrix as well as the distance through which water molecules diffuse in the film. These phenomena can effectively prevent the passage of water molecules and reduce the WVP.

Increasing the CIN dosage to 2.0% weakened the intermolecular forces within the composite membrane, indicating that the high concentration of CIN destroyed the network formed by CS and FG. Thus, CIN agglomerated on the membrane surface, which became rough and developed a scaly structure and cracks, resulting in a significant decline in the EB of the composite membrane. The decreased crystallinity of the composite film also reduced its gas-barrier performance.

## Conclusions

In this study, we investigated the effects of CIN addition on the properties of FG/CS composite films. As the CIN dosage increased, the TS, EB, and LT of the composite membrane gradually decreased. The WVP and OP increased in the initial stages and subsequently decreased. When the CIN content was 1.5%, CIN was uniformly distributed between FG and CS, thereby increasing the density of the network. Owing to the interactions between the three compounds, the state of the film changed from amorphous to crystalline, and the structure of the film was changed. CIN addition had no significant effect on the thermal stability of the composite membrane. These findings suggest that CIN is an effective additive to CS/FG films that can enhance their mechanical and chemical properties and significantly improve their applicability as food-grade packaging materials.

## Data Availability

All data generated or analyzed during this study are included in this manuscript.

## Abbreviations

FG: Flaxseed gum
CS: Chitosan
CIN: Cinnamaldehyde
TS: Tensile strength
EB: Elongation at break
LT: Light transmittance
WVP: Water vapor permeability
OP: Oxygen permeability
ATR-FTIR: Attenuated total reflectance Fourier-transform infrared
SEM: Scanning electron microscopy
TG: Thermogravimetry
DSC: Differential scanning calorimetry
TEO: Thyme essential oil
